# Analytical study of two feature extraction methods in comparison with deep learning methods for classification of small metal objects

**DOI:** 10.1186/s42492-022-00111-6

**Published:** 2022-05-10

**Authors:** Somaieh Amraee, Maryam Chinipardaz, Mohammadali Charoosaei

**Affiliations:** Department of Electrical and Computer Engineering, Jundi-Shapur University of Technology, Dezful, 64615/334, Iran

**Keywords:** Histogram of oriented gradients, Local binary pattern, Support vector machine, k-nearest neighbors, Deep learning

## Abstract

This paper addresses the efficiency of two feature extraction methods for classifying small metal objects including screws, nuts, keys, and coins: the histogram of oriented gradients (HOG) and local binary pattern (LBP). The desired features for the labeled images are first extracted and saved in the form of a feature matrix. Using three different classification methods (non-parametric K-nearest neighbors algorithm, support vector machine, and naïve Bayesian method), the images are classified into four different classes. Then, by examining the resulting confusion matrix, the performances of the HOG and LBP approaches are compared for these four classes. The effectiveness of these two methods is also compared with the “You Only Look Once” and faster region-based convolutional neural network approaches, which are based on deep learning. The collected image set in this paper includes 800 labeled training images and 180 test images. The results show that the use of the HOG is more efficient than the use of the LBP. Moreover, a combination of the HOG and LBP provides better results than either alone.

## Introduction

Object classification is one of the most important problems in the field of image processing and machine vision [1, 2]. In general, classification methods are divided into parametric and non-parametric methods. Parametric methods seek to extract parameters for describing a particular model based on a training-data analysis. After creating the desired model, it is possible to classify new samples [3, 4]. The support vector machine (SVM) and naïve Bayesian (NB) classifier are the most important parametric methods [5–9]. In parametric methods, a single model is considered for all of the input data. Although reducing the classification problem to finding a few finite parameters seems logical, this assumption may not be correct, potentially leading to a misdiagnosis of the output.

Non-parametric algorithms involve finding close or similar samples based on a suitable distance function, and then interpolating to find the correct output. The k-nearest neighbor (KNN) algorithm is one of the most popular non-parametric methods [10–12]. In non-parametric methods, there is no need to calculate the parameters in the training phase; however, an algorithm is designed by using training examples to classify new data. Because these methods are built directly on data instead of estimating or predicting parameters, they often achieve higher accuracy than parametric methods. Especially in cases where the distribution of the training data is such that it cannot be modeled with a finite number of parameters, the use of non-parametric methods seems more logical. The main drawback of non-parametric methods (including KNN) is that it is necessary to have all of the training samples to make decisions regarding a new sample. This leads to increased memory and computational costs, particularly for large datasets. If the training samples are expressed in the form of simple and short descriptors, it is possible to significantly avoid increasing the required memory volume and computational complexity.

Deep learning and deep networks have progressed in recent years and have achieved acceptable results in many applications, including object detection and recognition [4, 13–19]. Unlike traditional methods, these methods do not require a separate step to manually extract the necessary features.

One of the most well-known architectures proposed for object detection using deep learning [16] is “You Only Look Once” (YOLO) [20]. The importance of deep learning to various applications, such as those in agriculture, medicine, surveillance, and monitoring systems, is increasing daily [21–23]. The YOLO algorithm was first introduced in 2016, aiming to detect objects with a high speed and accuracy. This method introduced a new structure for object recognition systems. Owing to the significant attention it has received, different versions of YOLO have been implemented. YOLO stands for “You only look at the image once.” This term refers to the ability of the human visual system to detect objects at a glance. Therefore, the YOLO object recognition system is designed to provide a detection method that is similar to that of the human visual system. The YOLO algorithm consists of a 24-layer convolutional neural network (CNN) for feature extraction, and two fully connected layers for predicting the probabilities and coordinates of objects.

The faster region-based CNN (faster R-CCN) [24] is another state-of-the-art deep-learning-based technique. It was introduced in 2015 as a region proposal network (RPN) for sharing full-image convolutional features with a detection network, thereby enabling nearly cost-free region proposals. An RPN is a fully convolutional network that simultaneously predicts the bounding boxes and objectness scores at each position. It is the most widely used state-of-the-art version of the region-based CNN (R-CNN) [25].

The detection and classification of metal objects are important applications for machine image and vision processing, especially in industry and commerce [26–28]. In ref. [26], a deep CNN-based technique was proposed to detect metal screws and microdefects on their surfaces. Images of different types of metal screws were captured using industrial cameras. The proposed deep network architecture was then used to diagnose and check the flawlessness of the screw. The experimental results showed that this proposed technique could achieve a detection accuracy of 98%. However, this method was trained only on screw images, and did not include other categories of metal objects.

An automatic mobile recognition system for separating coins from banknotes was presented in ref. [27]. The proposed method was based on a scale-invariant feature transform color descriptor extraction method, and could be run on a smartphone. The results from this method were reviewed based on a set of images of banknotes and coins common in Jordan. Although this method had good accuracy in separating coins from banknotes, it seems that the problem (and method) resembled shape detection more than object classification. Because the coins were all circular and the banknotes were rectangular, the proposed method was not generalizable to detect other objects.

The method proposed in ref. [28] focused on a screw identification system for use in various industries, particularly in the automotive industry. The proposed method was based on a back-propagation neural network. The results from the experiments showed that the system could detect moving objects on a production line with appropriate accuracy; however, it was limited to detecting only two categories (screws and nuts).

One of the most critical challenges in object classification is choosing an appropriate feature-extraction method [13]. An improper selection at this stage can affect the accuracy of classification, and lead to errors in decision-making. Accordingly, it is necessary to conduct comprehensive research on the efficiency of various feature-extraction methods. A feature extraction method may work well with a particular classification algorithm, but may not work very well if used in another classifier. Thus, it is necessary to compare the results from several classification algorithms to conduct a detailed analytical study. In view of the above, the main purpose of this study is to comprehensively compare the performances of the histogram of oriented gradients (HOG) and local binary pattern (LBP) feature vectors.

The present study addresses the efficiency of the HOG and LBP approaches in classifying four groups of metal objects (screws, nuts, keys, and coins). To study these features in detail, three different classification methods are employed, i.e., the non-parametric KNN algorithm, SVM, and NB methods. The accuracy of each method is investigated using the HOG and LBP feature vectors, along with a combination of these two feature vectors. Then, the effectiveness of these methods is compared with those of the YOLO and faster R-CNN deep learning methods.

The major contributions of this study include (1) providing an analytical study of the use of HOG and LBP methods for the classification of small objects; (2) using three conventional methods (KNN, SVM, and NB) for a comprehensive comparison of the HOG and LBP; (3) using YOLO as a deep-learning method for a more detailed comparison; and (4) providing a diverse image set of metal objects able to be used in future research as a benchmark set for comparing different methods.

The following section discusses the methods in the proposed structure. The results and discussion section analyzes the obtained results from traditional techniques and deep learning methods, e.g., YOLO and faster R-CNN. The last section presents the conclusions of this study.

## Methods

**Fig. 1:**
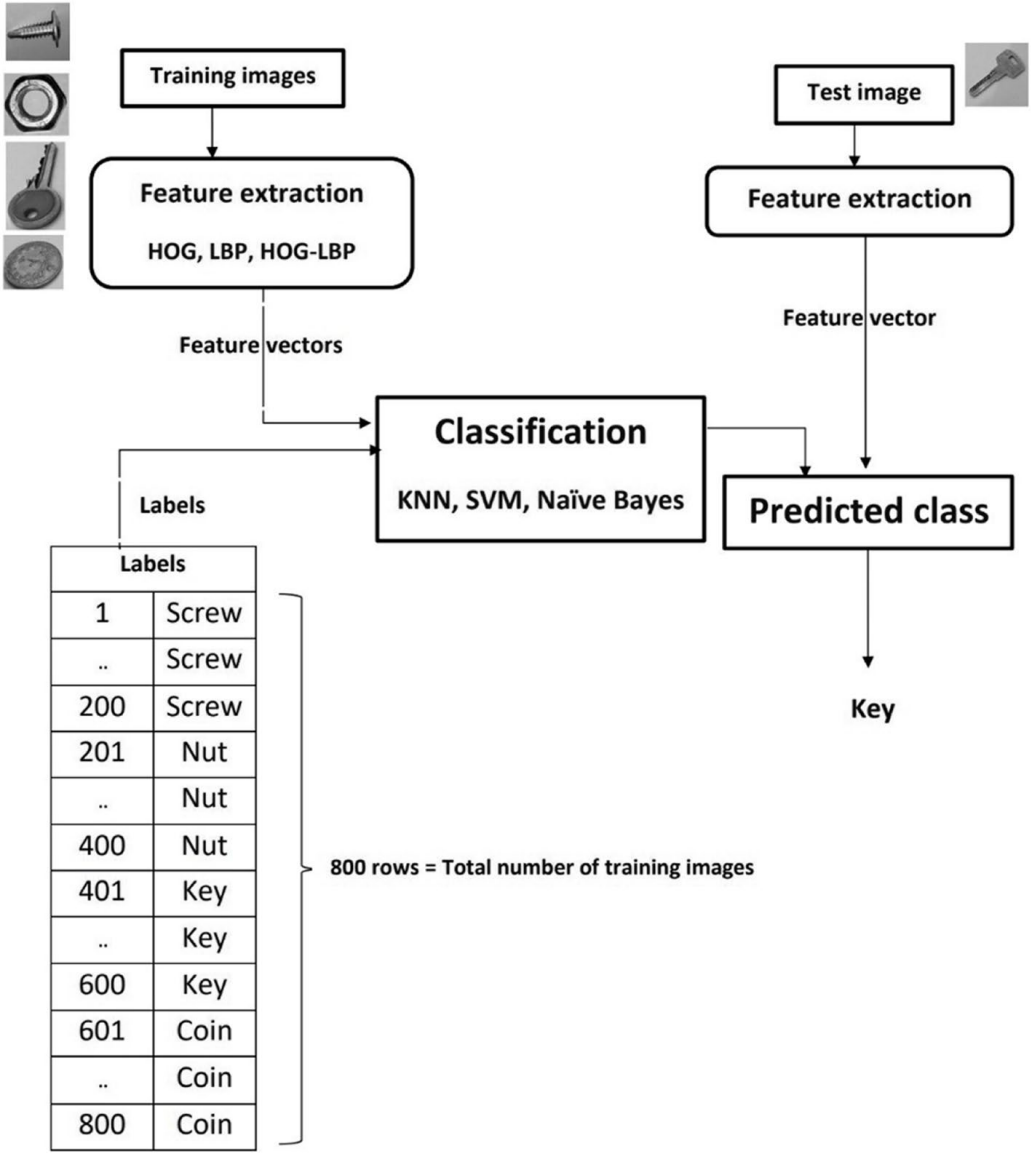
General structure of the proposed method, including HOG, LBP, KNN, SVM, and NB approaches

Figure 1 shows the general structure of the proposed method for image classification. By extracting the corresponding descriptors for each training sample, all of the training data are in the form of a matrix in which each row represents the feature vector of a sample, and each column represents a feature in the feature space. The training data were labeled; therefore, each related class is available at the start of the proposed algorithm. A parametric or non-parametric model is created based on these training samples for predicting the class of a test image. As shown in Fig. 1, feature extraction involves the use of one of the HOG or LBP methods, or of a combination of these two methods. Classification refers to one of the three methods mentioned above: KNN, SVM, and NB. With the arrival of new samples (test images), the desired features are extracted, and the label of the unknown sample is estimated using the created model. After the test samples are determined, a confusion matrix is generated for each classification model. The efficiencies of the different methods are examined using the confusion matrices.

### HOG

The HOG was introduced in 2005 for pedestrian detection in static images. Today, this method plays an important role in identifying humans in movies [29, 30], as well as in various other applications such as sketch-based image retrieval [31] and real-time vehicle detection [32].

To extract the HOG, an image is first filtered using the horizontal and vertical operators in the *x*- and *y*-directions, so that image gradients are obtained for the *x*- and *y*-directions.

1 $${G}_x={\mathrm{D}}_x\ast I$$

2 $${G}_y={\mathrm{D}}_y\ast I$$

3 $${D}_y=\left[\begin{array}{c}-1\kern0.75em \\ {}0\\ {}1\end{array}\right],{D}_x=\left[-1\kern0.75em 0\kern1em 1\right]$$

In the above, *I* is the original image. D*x* and D*y* are the filter masks in the *x* and *y* directions, respectively, and are defined as the vectors of Eq. (3). In ref. [29], more complex masks, including Sobel operators, were used to calculate the image gradients, and their performance was evaluated. This study reveals that the use of the masks of Eq. (3), in addition to providing simplicity, leads to better results in pedestrian detection. In Eq. (3), G*x* and G*y* denote the gradients of the image in the *x* and *y* directions, respectively, and the sign * indicates the convolution operation. In convolution operations, the neighbors of a central pixel are summed with specified weights, and the result is placed as the current pixel value. The weights are determined by a weight matrix or convolution mask. After calculating G*x* and G*y*, the magnitude and orientation of the gradient in each pixel are obtained as follows:

4 $$\left|\mathrm{G}\left(\mathrm{i},\mathrm{j}\right)\right|=\sqrt{{\left[{\mathrm{G}}_{\mathrm{x}}\left(\mathrm{i},\kern0.5em \mathrm{j}\right)\right]}^2+{\left[{\mathrm{G}}_{\mathrm{y}}\left(\mathrm{i},\kern0.5em \mathrm{j}\right)\right]}^2},\kern0.5em {\uptheta}_{\mathrm{G}}{=\tan}^{\hbox{-} 1}\left[\frac{{\mathrm{G}}_{\mathrm{y}}\kern0.5em \left(\mathrm{i},\kern0.5em \mathrm{j}\right)}{{\mathrm{G}}_{\mathrm{x}}\left(\mathrm{i},\kern0.5em \mathrm{j}\right)}\right]$$

Here, |G| is the magnitude of the gradient, θ is the gradient direction, and i and j represent rows and columns in the image, respectively. To calculate the gradient histogram in each cell, the gradient orientation is first limited to a range of 0–180, as follows:

5 $${\uptheta}_{\mathrm{G}}^{\prime }=f(x)=\left\{\begin{array}{c}{\uptheta}_{\mathrm{G}},\kern5.25em 0\le {\uptheta}_{\mathrm{G}}<180{}^{\circ}\\ {}{\uptheta}_{\mathrm{G}}-180,\kern0.5em 180{}^{\circ}\le {\uptheta}_{\mathrm{G}}\le 360{}^{\circ}\end{array}\right.$$

To calculate the histogram of gradients, the distance between 0–180° is divided by *n* equal distances, representing the number of directions of the gradient or histogram bars. Each of these distances forms a histogram channel. The range from 0–180° is used instead of the 360° range because usually, additional bars are needed for extraction in the range of 0–360°. Thus, the smaller range saves more time for feature extraction. Experimental observations have also shown that using a 360° range has little effect on improving the results relative to a 180° range. As discussed in ref. [29], a nine-bar histogram achieved better results in experiments; accordingly, the present study uses the same number of bars to calculate the HOG.

**Fig. 2:**
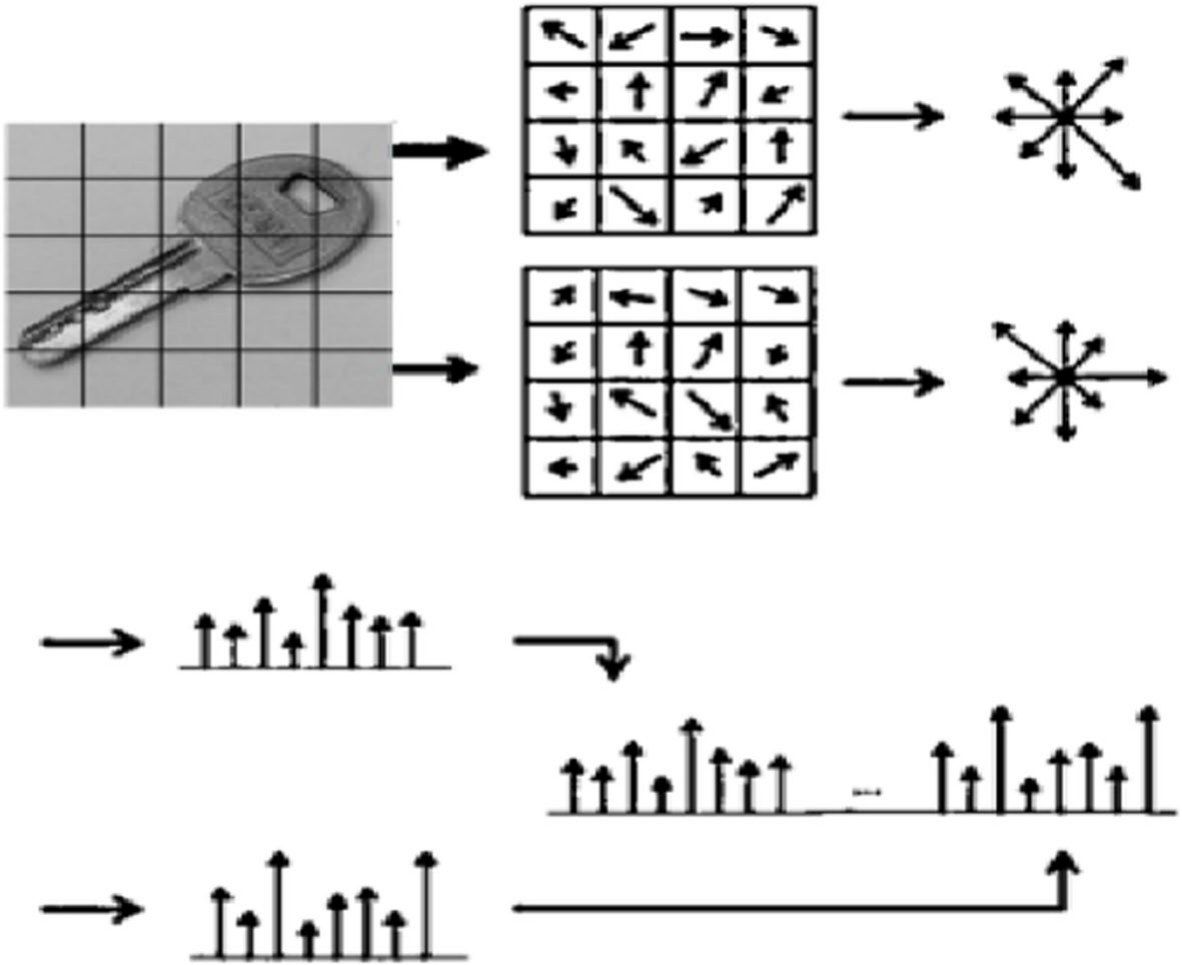
HOG

To calculate the histogram, the image is divided into several cells. Each pixel then votes for one of the histogram channels, based on its gradient orientation. These votes are weighted based on the magnitude of the gradient in that pixel. This generates a histogram for each cell for describing the gradient of the pixels. In some cases, the HOG is calculated for a block (consisting of several cells) by connecting the histograms of adjacent cells (Fig. 2).

### LBP

The LBP is another type of visual descriptor used in various machine vision applications [33–35]. This descriptor can be used for powerful feature extraction methods for face descriptions [36], analyzing wear in carpets [37], etc.

The feature vector in the LBP for gray-scale images is calculated as follows.

**Fig. 3:**
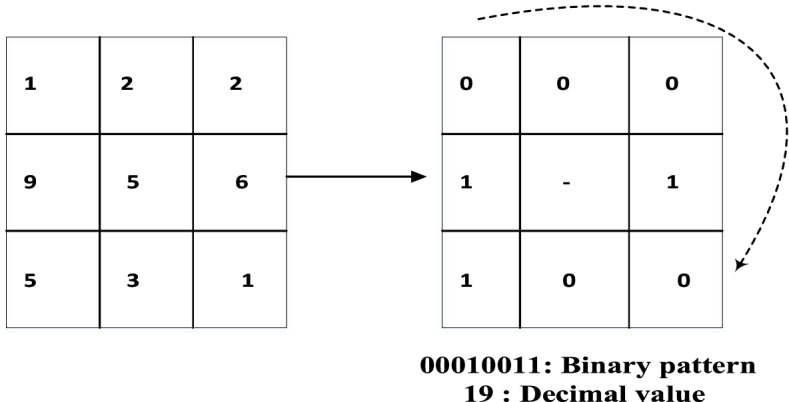
LBP

- The desired image is divided into several blocks, and each block is divided into several cells.
- The following calculations are performed for each pixel in a cell.
- Each pixel is compared to its eight neighboring pixels. The neighboring pixels are examined individually in a particular direction (e.g., clockwise).
- When the center pixel is larger than the neighboring pixel, the number ‘0’ is written; otherwise, the number ‘1’ is written. In this manner, an eight-bit number is obtained by comparing the central pixel with its eight neighbors. For convenience, this number is usually converted to a decimal number between 0–255 (Fig. 3).
- A histogram of the numbers obtained in the previous step is calculated for each cell. This histogram has 256 bars (from 0–255), and each bar shows the number of repetitions of a specific number in that cell.
- If necessary, the desired histogram is normalized.
- The histogram of the entire block is obtained by connecting the histograms of neighboring cells. Thus, if a block contains four cells, the generated feature vector has a length of 256 × 4.

Figure 3 shows an example of binary-pattern calculations in a 3 × 3 neighborhood. The binary pattern 00010011 is assigned to the central pixel, with a gray level of ‘5’ in the image on the left. After completing the calculations for all of the blocks, the generated feature vectors are processed by using an appropriate model to categorize the desired images. These classifiers can be used to categorize objects, recognize faces, analyze textures, and so on.

Different types of LBP algorithms have been proposed, with various changes relative to the original algorithm. One of the most useful and widely used types of LBP is the uniform pattern, which can significantly reduce the length of the feature vector [35]. This idea stems from the fact that the number of occurrences of certain binary patterns, called uniform binary patterns, is particularly important.

A generated binary pattern for a pixel is called a uniform pattern if it has a maximum of two 0–1 or 1–0 transitions. For example, 00010000 is a uniform pattern with two transitions, 0–1 and 1–0, but pattern 01010111, with five transitions, is not uniform. Uniform binary patterns with the highest number of events correspond to the basic features of the image, such as its edges, corners, and important points [34].

Therefore, uniform patterns can be considered as factors in identifying the main features of an image. All non-uniform patterns are assigned to a single bin, and each uniform pattern has a separate bin. As 58 uniform patterns are in the range of 0–255, the uniform LBP feature vector will have a length of 59; this is significantly reduced from the length of 256 in an ordinary LBP.

In this manner, by comparing local neighborhoods and calculating a uniform LBP histogram, an image signature is created to represent the type of texture. The generated signatures are sufficiently distinctive for images falling into different classes. Therefore, the LBP can be used to classify the textures.

### KNN algorithm

The KNN algorithm is one of the most common non-parametric classification methods [10–12, 38]. In non-parametric methods, there is no need to calculate the parameters in the learning phase. Using the data itself, an algorithm is designed to check whether the new data belong to the training class. The advantage of these methods is that they do not require parameter estimations, and are usually more accurate than parametric methods; however, their main limitation is that they require all of the training samples to classify new samples. This increases the memory and computational costs, especially for large datasets. The KNN classification steps used to categorize images are as follows.

- The training datasets and related labels are uploaded, and then a value of K is chosen as the number of neighbors.
- The distance between the test image and each training sample is calculated.
- The training samples are sorted in ascending order, based on the distances calculated in the previous step.
- The first K items are selected from the sorted set.
- The label is checked for the selected items in the previous step.
- The most frequently labeled class is selected as the predicted class for the test sample.

**Fig. 4:**
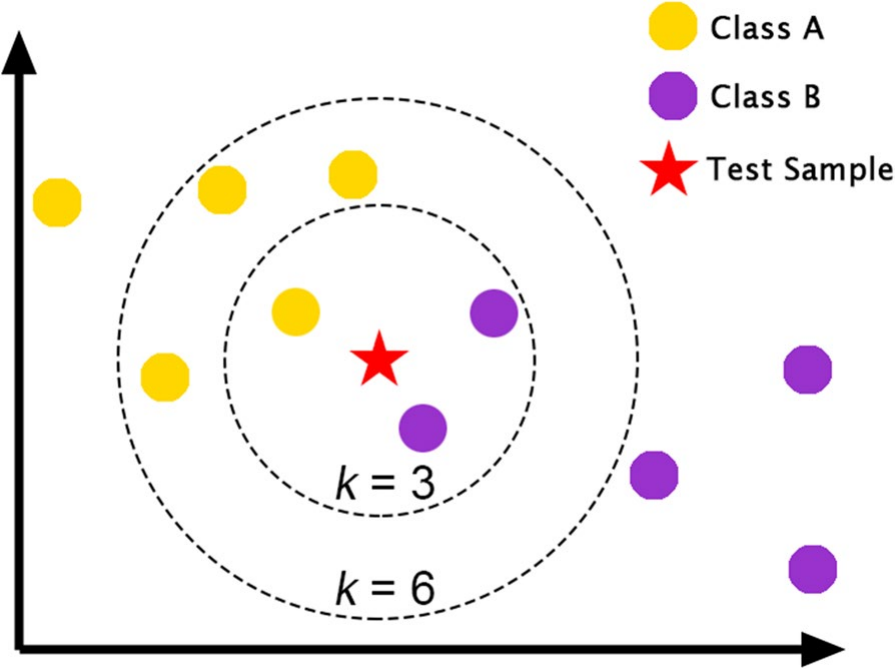
Two-class classification using the KNN algorithm

Figure 4 shows an example of two-class classification using the KNN algorithm. In this case, if *k* = 3, the test sample is considered as being in class B, because out of the three close neighbors, two neighbors are labeled B, and one neighbor is labeled A.

If *k* = 6, the situation will be different because in this case, four neighbors are labeled A and two neighbors are labeled B; therefore, the test sample is placed in class A. The accuracy of the classification algorithm can be verified by comparing the predicted labels with the actual labels of the test samples.

### SVM-based classifier

**Fig. 5:**
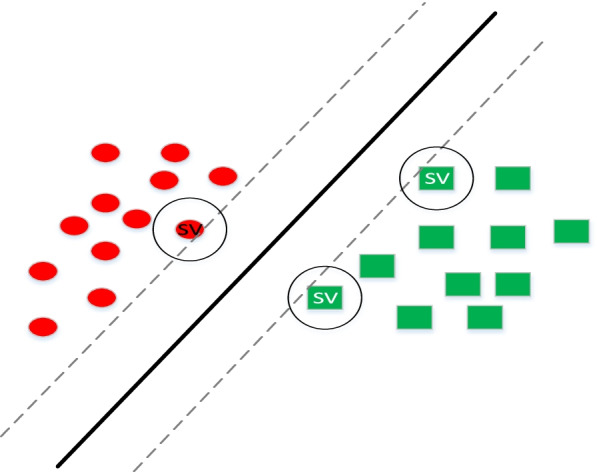
SVM

The SVM is one of the currently widely used methods for classification [5, 6]. The current popularity of the SVM method can be compared with the popularity of neural networks over the past decade. The SVM is based on a linear classification of data. Figure 5 shows an example of a dataset that can be categorized linearly. Several lines are drawn to categorize the data. In a linear division of data, an attempt is made to select a line with a more reliable margin. Quadratic programming is used to find the optimal linear separator; this is a known method for solving limited problems.

The basic idea of the SVM is that, assuming that the categories are linearly separable, a line is obtained with a maximum margin to separate the categories. To find such a separator, two boundary lines are drawn parallel to the separator line, and are separated such that they collide with the data. The separation that maximizes the training data margin among the linear separators minimizes the generalization error. The training data closest to the separating line are called the support vectors (Fig. 5). Notably, for dimensions greater than two, the term ‘hyperplane’ is used instead of ‘line.’ A hyperplane is a geometric concept representing a generalization of the concept of a plane in *n*-dimensions. In other words, the hyperplane defines a subsequent *k* subspace in an *n*-dimensional space such that *k* < *n*.

One of the best properties of SVM is that, in cases where the data are not linearly separable, the SVM maps the data to a larger dimension using a nonlinear mapping function Φ. In this way, the data can be linearly separated in this new space.

**Fig. 6:**
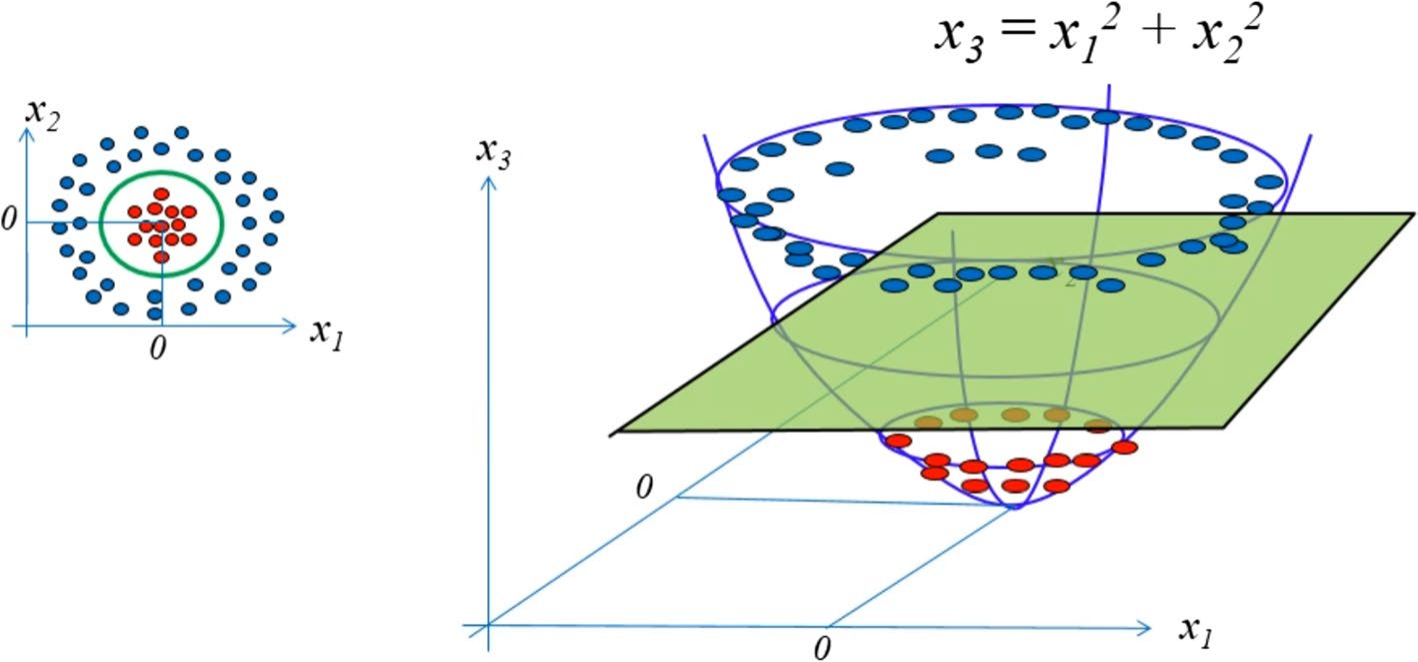
Mapping from two-dimensional space to three-dimensional space

This implies that samples that are not linearly separable in their original space *I* move to a new feature space called F to create a hyperplane for separating them. When this hyperplane returns to its original space *I*, it forms a nonlinear curve. As shown in Fig. 6, the input data are not linearly separable, and no line can accurately represent the boundary between the two classes. However, by mapping them from a two-dimensional space to a three-dimensional space, it is possible to create a hyperplane for separating the boundaries of these two classes.

### NB categories

NB is a probability-based machine-learning algorithm that can be used for a wide range of classification problems [7–9]. Common applications of the NB algorithm include spam filtering, document classification, and emotion prediction.

This NB algorithm uses the Bayesian theorem to produce results, based on a hypothesis of strong independence between the features. This implies that changing the value of one feature does not directly affect the value of any other feature. Although this assumption is simplistic (as the algorithm’s name implies) for real-world datasets, the NB classifier has nevertheless found a worthy place among classification algorithms.

Suppose ***X*** = (*x*_1_, *x*_2_, .. *x*_*n*_) expresses a data sample as a vector of *n* independent variables. To calculate the probability *P*[*C*_*k*_| (*x*_1_, *x*_2_, .. *x*_*n*_)], it is sufficient to use the joint probability, and to simplify it using a conditional probability concerning the independence of the variables.

### Deep learning methods

YOLO is a state-of-the-art real-time object detection system. It uses a single neural network to obtain a full image. This network divides the image into regions, and predicts bounding boxes and probabilities for each region. These bounding boxes are weighted using the predicted probabilities.

To use the YOLO algorithm, it is first necessary to prepare training images.

Preparing a training set for the YOLO algorithm is different from the approach for traditional methods, such as SVM or KNN. It is necessary to draw a bounding box around each object, and the corresponding class is determined by the user. Various programs can be used to draw bounding boxes and tag them. In this study, the MAKESENSE (https://www.makesense.ai/) program is used to tag the training images.

**Fig. 7:**
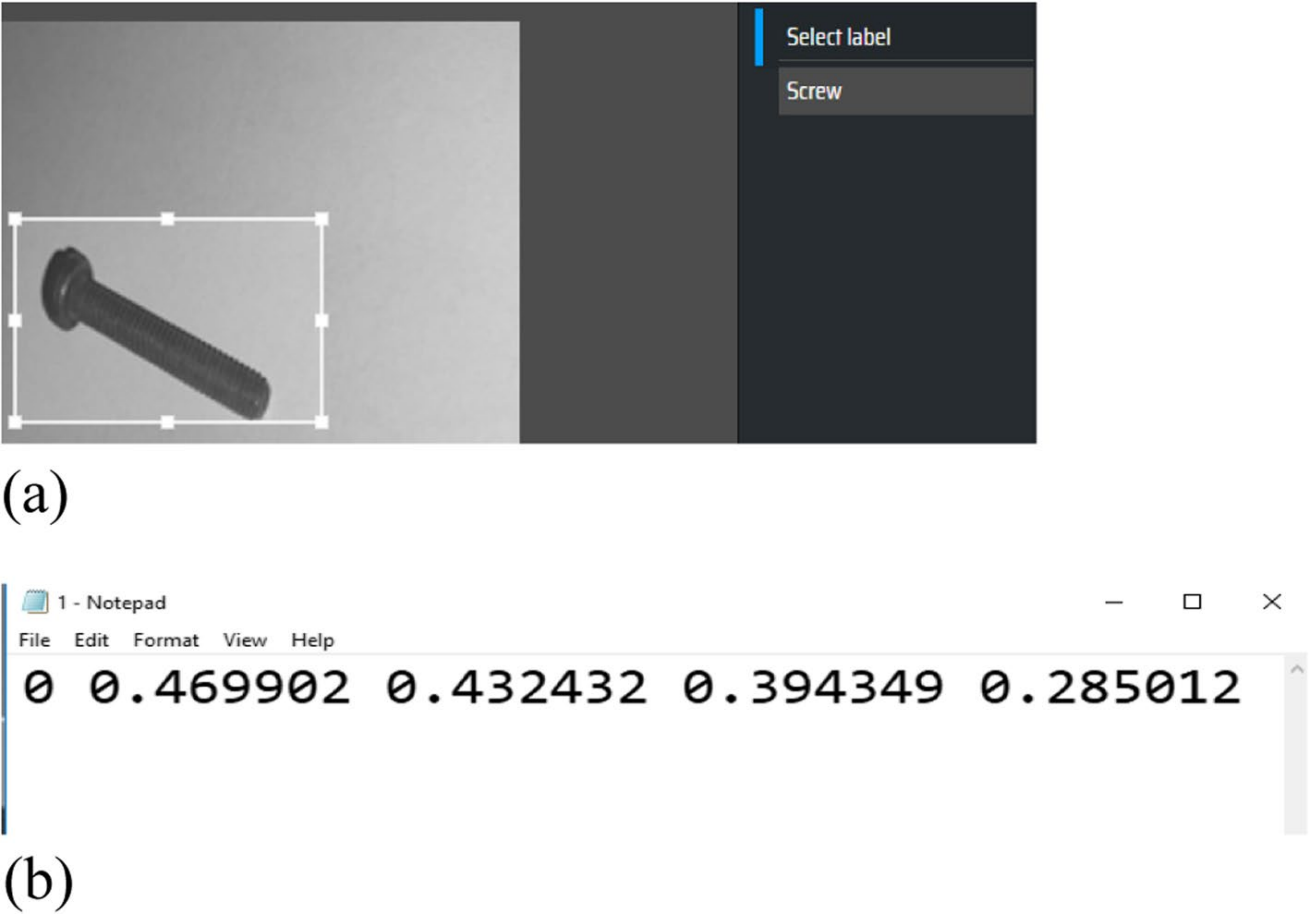
Preparing training data set for deep learning methods. (**a**): Labeling training images; (**b**): Output text file

In this manner, a text file is created for each of the training images, in which the specifications of an object are written in each row. The first number is related to the class of the object; then the coordinates of the center of the rectangle and its length and width are written. The number of rows in the file is equal to the number of objects in the image. Figure 7 shows an example of the training data and generated file from the output of the MAKESENSE program. This program is also used to tag images for another deep learning method (faster R-CNN).

Faster R-CNN is a deep convolutional network used for object detection, and works as a single, end-to-end, unified network for the user. The network can predict the locations of multiple objects in a short time. Researchers at UC Berkeley developed R-CNN [25] in 2014. The R-CNN is a deep convolutional network capable of detecting 80 different types of objects in images. In comparison with the generic pipeline for the object detection methods, the foremost contribution of R-CNN is the extraction of features based on a CNN.

The R-CNN consists of three principal modules. The first module produces 2000 region proposals using a selective search algorithm. After resizing to a fixed predefined size, the next module extracts a feature vector of length 4096 from each region proposal. The third module utilizes a pre-trained SVM algorithm to classify the region proposal into one of the object classes, or as background. The R-CNN model has some weaknesses: it is a multistage model, where each stage is an independent part. Consequently, it cannot be trained end-to-end. It captures the extracted features from a pre-trained CNN on the disk to train the SVMs. This requires a bulk storage on the order of gigabytes. The R-CNN depends on a selective search algorithm for creating region proposals, which takes a long time. In addition, this algorithm cannot be customized for detection problems. Each region proposal is fed without dependence on the CNN for the feature extraction, making it inappropriate to run the R-CNN in real time. As an extension of the R-CNN model, the fast R-CNN model was proposed [24] to overcome some of these limitations.

## Results and Discussion

In this study, conventional classification methods (SVM, KNN, and NB) were implemented using MATLAB software version 2019. A set of images, including 800 training images and 180 test images, was prepared to analyze the efficiency of the HOG and LBP methods. More specifically, 200 training and 45 test images were considered for each of the four categories (screws, nuts, keys, and coins).

**Fig. 8:**
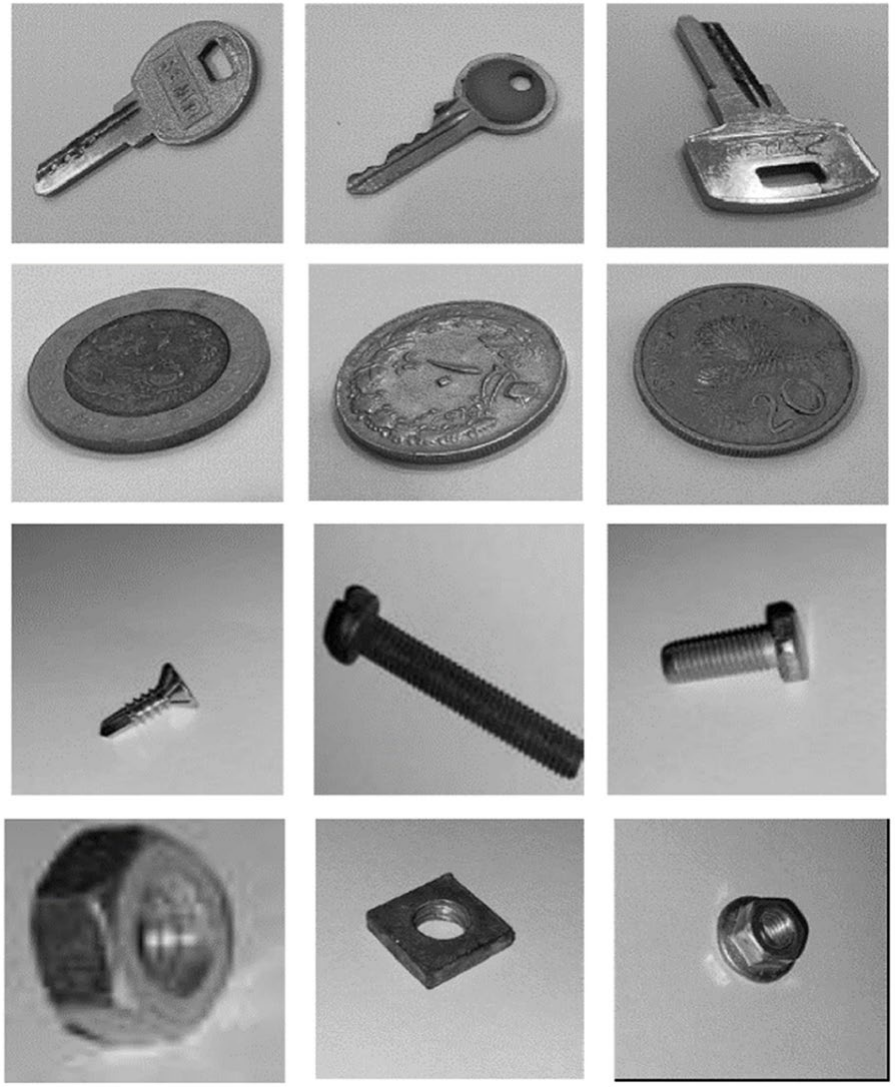
Examples of images in the collected dataset

In the case of the HOG, the size of each cell was 64 × 64 pixels, each block contained 2 × 2 cells, the number of bins in the orientation histograms was 10, and the length of the HOG feature vector was 360. For the LBP, the cell size was 64, and a rotationally invariant uniform feature vector with a length of 160 was employed. Thus, the combined feature vector (HOG-LBP) had a length of 520. There was an attempt to provide sufficient variety in terms of the types of objects and lighting conditions. Figure 8 shows examples of images in the collected dataset.

Table 1 lists the confusion matrices obtained using the SVM method. This table has three matrices, from top to bottom, corresponding to the states of the HOG, LBP, and a combination of them (HOG-LBP). The KNN-based classification and NB-based classification results are shown in Tables 2 and 3, respectively. As shown in Table 1, when using the SVM-based classification, the feature extraction by the LBP method performed worse than that by HOG for detecting keys and coins, but performed better than the HOG for detecting nuts.

**Table 1:** Confusion matrix in the SVM classifier

Method	Confusion matrix				
HOG					
		Screw	Nut	Key	Coin
	Screw	35	6	3	1
	Nut	5	34	0	6
	Key	4	1	39	1
	Coin	1	7	0	37
LBP					
		Screw	Nut	Key	Coin
	Screw	35	5	0	5
	Nut	4	36	2	3
	Key	1	2	38	4
	Coin	2	11	3	29
HOG-LBP					
		Screw	Nut	Key	Coin
	Screw	40	4	0	1
	Nut	1	42	0	2
	Key	1	0	44	0
	Coin	1	4	0	40

**Table 2:** Confusion matrix in the KNN classifier

Method	Confusion matrix				
HOG					
		Screw	Nut	Key	Coin
	Screw	39	3	1	2
	Nut	4	36	0	5
	Key	3	0	42	0
	Coin	1	4	0	40
LBP					
		Screw	Nut	Key	Coin
	Screw	37	4	1	3
	Nut	8	30	1	6
	Key	2	2	37	4
	Coin	6	7	1	31
HOG-LBP					
		Screw	Nut	Key	Coin
	Screw	40	4	0	3
	Nut	5	36	0	7
	Key	4	0	39	1
	Coin	0	1	0	40

**Table 3:** Confusion matrix in the NB classifier

Method	Confusion matrix				
HOG					
		Screw	Nut	Key	Coin
	Screw	28	13	3	1
	Nut	9	28	0	8
	Key	2	1	42	0
	Coin	3	8	0	34
LBP					
		Screw	Nut	Key	Coin
	Screw	17	22	0	6
	Nut	6	20	0	19
	Key	5	15	8	17
	Coin	1	7	0	37
HOG-LBP					
		Screw	Nut	Key	Coin
	Screw	25	17	2	1
	Nut	5	30	0	10
	Key	6	0	39	0
	Coin	0	9	2	34

**Fig. 9:**
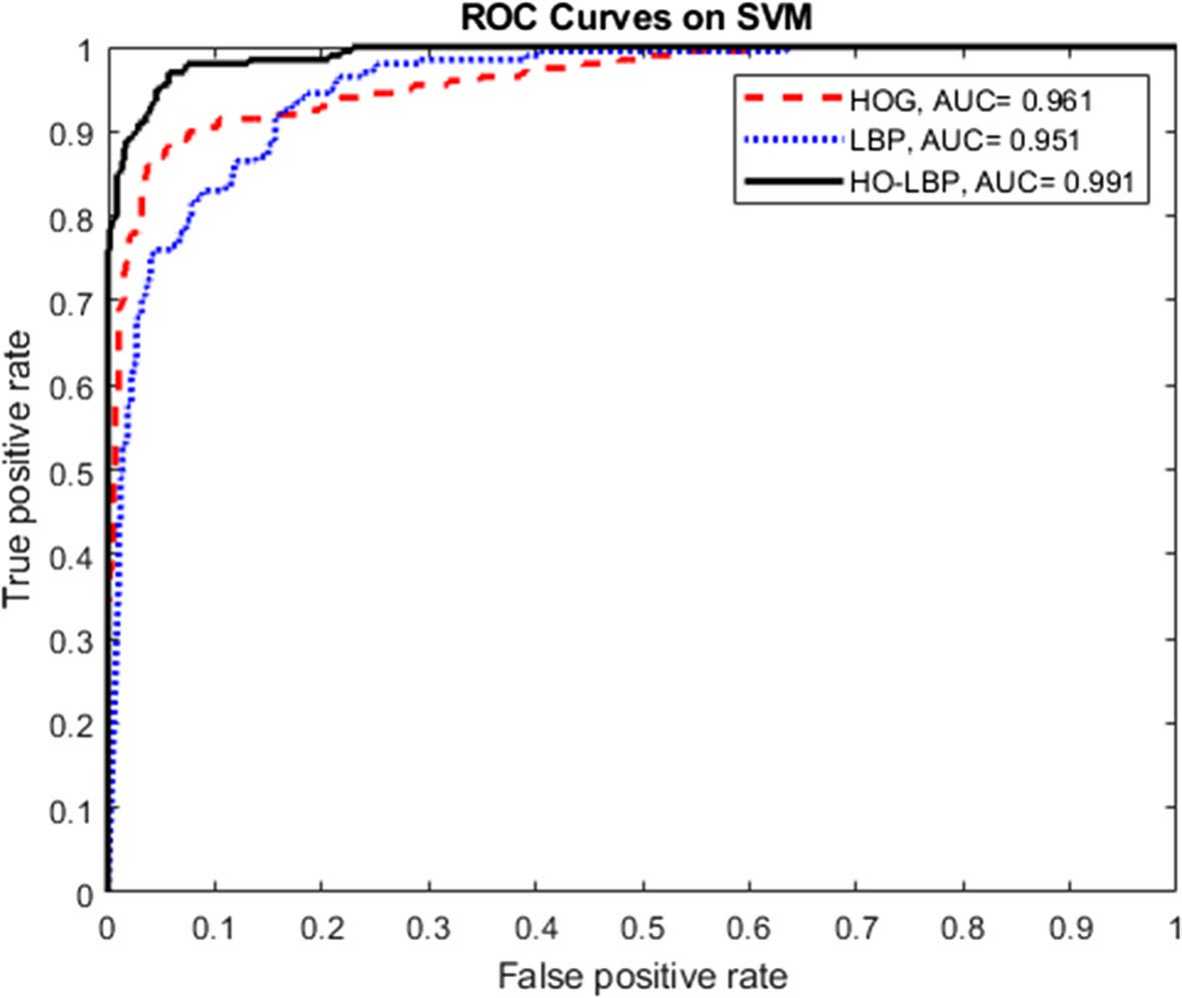
ROC curves of SVM

In detecting the nuts, using the HOG results in 34 correct detections, but this value is 36 for the LBP. This table also shows that using the HOG-LBP feature vector combination, which results from the connection of two separate feature vectors, yields better results than the independent use of either of these features. Figure 9 shows the receiver operating characteristic (ROC) curves of the SVM and area under the curve (AUC) values for the three different feature vectors. This figure shows that the combined feature vector (HOG-LBP) achieves better results than the independent use of the HOG and LBP.

Table 2 presents the results for the KNN classifier. In the case of the KNN algorithm, choosing an appropriate value for *k* may affect the efficiency of the classification. The value of *k* = 3 achieved a greater accuracy for metal object classification in the experiments. The matrices in this table indicate that using the HOG leads to better results than using the LBP. As shown in this table, using the HOG-LBP combination to detect three classes (i.e., screws, nuts, and coins) shows better results than using each of them separately. However, the use of the HOG-LBP feature performs worse than the independent use of the HOG in the case of the key class (39 vs 42).

An examination of Table 3 shows that HOG performs better than the LBP in the case of the NB classifier. This table also indicates that the HOG-LBP combination does not achieve a higher accuracy than the HOG and LBP separately when using am NB classifier.

**Fig. 10:**
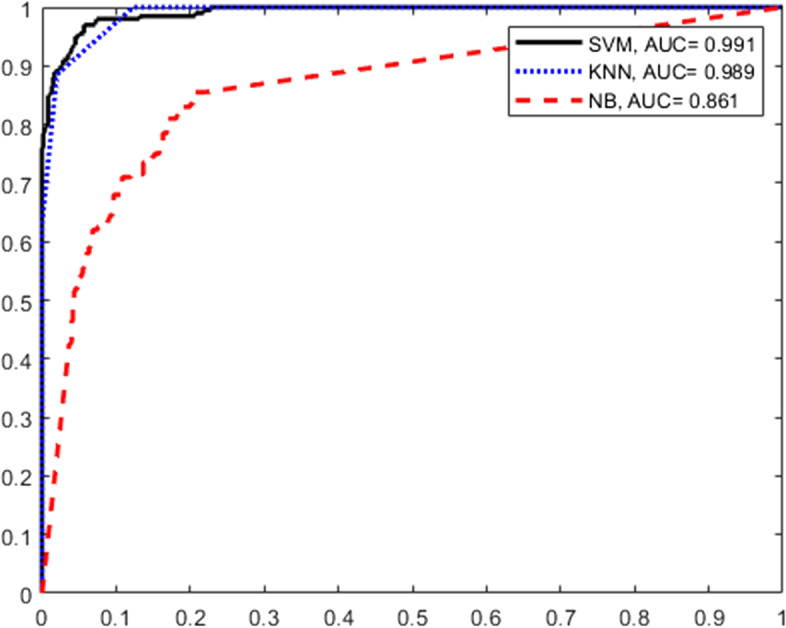
ROC curves for SVM, KNN, and NB

Figure 10 depicts the ROC curves for the SVM, KNN, and NB approaches, along with the AUC values. As shown, the SVM classifier achieves better results than the KNN and NB classifiers. Therefore, from Figs. 9 and 10, it can be concluded that using the SVM classifier for the HOG-LBP vector provides the best result for the classification of small metal objects when using conventional methods. In the following, the effectiveness of two deep learning methods is examined: YOLO and faster R-CNN.

**Fig. 11:**
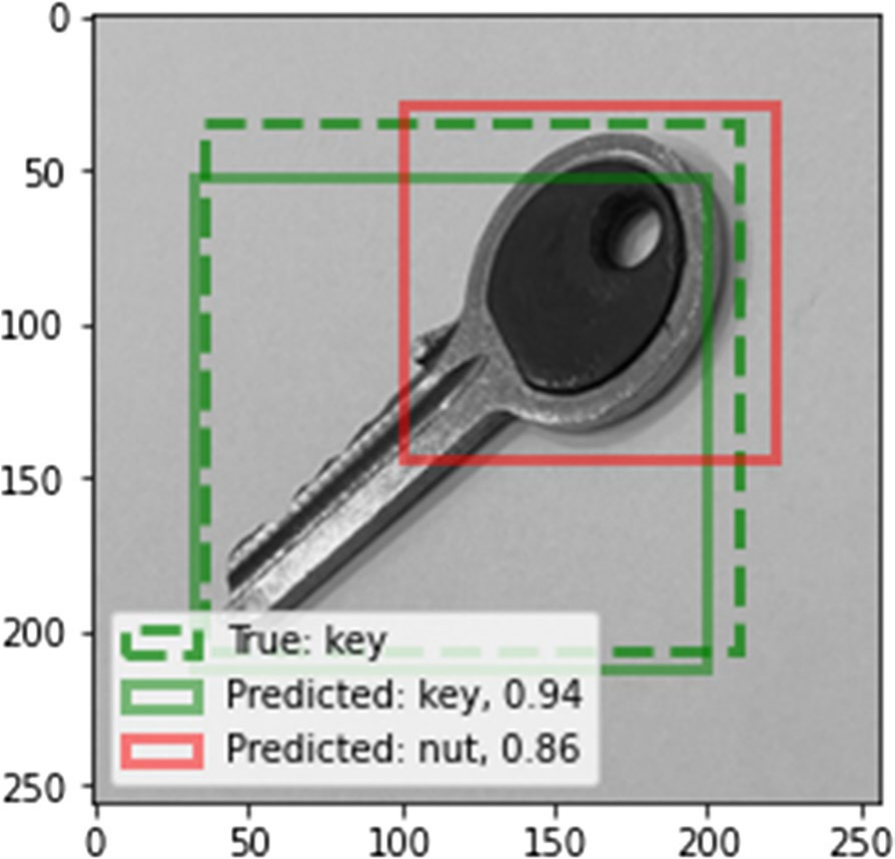
False classification in faster R-CNN

**Fig. 12:**
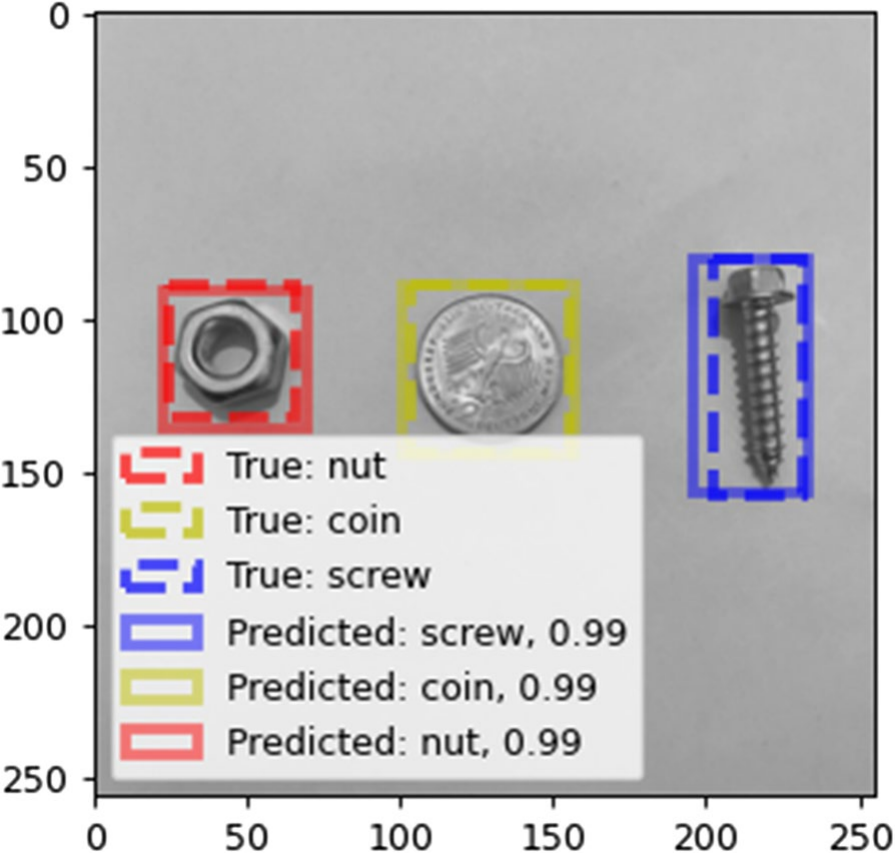
Multi-object detection in the deep learning methods

In implementing the deep learning method, 10% of the training images were employed as a validation set. Table 4 presents the confusion matrix obtained using YOLO version 5 and faster R-CNN. As shown in Table 4, the classification achieves high accuracy with these two methods. In the case of YOLO, one nut is not placed in any of the classes. In the case of faster R-CNN, one nut is placed in an incorrect class (coin) and part of one key is recognized as a nut, as can be seen from Fig. 11. One of the advantages of deep learning methods such as YOLO and faster R-CNN is that they are able to identify the locations of the objects as well as classify them in multi-object conditions, i.e., where there are several objects in one frame. In contrast, in traditional methods, this capability does not exist, and it is necessary to use an auxiliary algorithm to detect the location of an object. An example of multiple detections is shown in Fig. 12.

**Table 4:** Confusion matrix of the deep learning methods

Method	Confusion matrix				
YOLO					
		Screw	Nut	Key	Coin
	Screw	45	0	0	0
	Nut	0	44	0	0
	Key	0	0	45	0
	Coin	0	0	0	45
Faster R-CNN					
		Screw	Nut	Key	Coin
	Screw	45	0	0	0
	Nut	0	44	0	1
	Key	0	1	45	0
	Coin	0	0	0	45

Notably, the YOLO and faster R-CNN algorithms do not require a separate step to extract the feature vectors. In this respect, they may be superior to feature-based methods such as KNN and SVM. However, implementing a deep learning algorithm has more hardware requirements than feature-based extraction methods. In the present study, the YOLO and faster R-CNN algorithms were implemented using Google Colab. Another drawback of YOLO and faster R-CNN is the method for labeling the training data, which makes the training phase of these algorithms more difficult and time-consuming than conventional methods.

Examination of the confusion matrices in Tables 1, 2, 3 and 4 indicates that generally, feature extraction in the HOG method performs better than that in the LBP method, but their combination feature vector (HOG-LBP) is more accurate than their independent use for classifying small metal objects. In addition, the SVM-based method performs better than the KNN and NB-based methods, and the NB-based method achieves lower accuracy than the other methods. Although the SVM-based method is less accurate than YOLO and faster R-CNN, it has fewer hardware requirements and an easier training phase than deep learning methods.

## Conclusions

In this study, the efficiency of two feature extraction methods (the HOG and LBP) in classifying small metal objects including screws, nuts, keys, and coins was evaluated. Three different classifications, including KNN-, SVM-, and NB-based methods were used. The experiments indicate that generally, the HOG is better than the LBP, and that using their combination feature vector (HOG-LBP) is better than using each separately. The effectiveness of these two methods was also compared with those of YOLO and faster R-CNN, which are based on deep learning. Although deep learning methods do not require a separate step for feature extraction, they require more powerful platforms than traditional methods.

The strength of conventional feature extraction methods, such as the HOG and LBP, is that the extracted feature vector can be used in different classifiers to select a more accurate classifier. However, the disadvantage of these methods is that they can only classify detected objects. In other words, they cannot detect the positions of foreground objects in an image. In such a situation, a foreground extraction method must be applied, and then a traditional method (such as the HOG) can be used to produce the feature vector for each foreground region. In contrast, deep learning methods determine object positions in an image, as well as classifying those objects. This is the most positive point of the YOLO and faster R-CNN algorithms, but their disadvantage is that they require manual image annotation in the training phase, which is time-consuming and laborious for a large dataset. Nevertheless, the importance of deep learning methods is increasing in various applications, such as agriculture, medicine, and surveillance systems.

In continuing the research done in this study, other texture feature analyses, such as the Gabor filter, can be examined. Four-class classification can be developed to classify with greater numbers. In addition, ensemble methods for considering various sizes of objects can be employed. A comparison with other deep learning methods will provide a better understanding of the efficiency of the different feature extraction methods. In general, the results of this study can be applied to factories and industrial workshops. Therefore, obtaining industrial images and providing practical implementations of the proposed method in real environments can be explored in future studies.

## Data Availability

The datasets used and/or analyzed during the current study are available from the corresponding author upon reasonable request.

## Abbreviations

HOG: Histogram of oriented gradients
LBP: Local binary pattern
KNN: K-nearest neighbors
YOLO: You Only Look Once
SVM: Support vector machines
CNN: Convolutional neural network
RPN: Region proposal network
R-CNN: Region-based convolutional neural network
NB: Naïve Bayesian
ROC: Receiver operating characteristic
AUC: Area under the curve
